# Circulating IFNγ-associated protein signatures predict response to neoadjuvant immunotherapy in patients with stage III melanoma

**DOI:** 10.1186/s12935-025-04067-4

**Published:** 2025-11-25

**Authors:** Fei Yang, Su Yin Lim, Ines Pires da Silva, Lijia Yu, Jordan W. Conway, Alexander M. Menzies, Georgina V. Long, Jean YH Yang, Helen Rizos

**Affiliations:** 1 Macquarie Medical School, Faculty of Medicine, Health and Human Sciences, Macquarie University, Sydney, NSW 2109 Australia; 2 Melanoma Institute Australia, The University of Sydney, Sydney, NSW Australia; 3 School of Mathematics and Statistics, The University of Sydney, Sydney, NSW Australia; 4 Sydney Precision Data Science Centre, The University of Sydney, Sydney, NSW Australia; 5 Blacktown Hospital, Sydney, NSW Australia; 6 Faculty of Medicine and Health, The University of Sydney, Sydney, NSW Australia; 7 Charles Perkins Centre, The University of Sydney, Sydney, NSW Australia; 8 Royal North Shore and Mater Hospitals, Sydney, NSW Australia

**Keywords:** Immune checkpoint inhibitors, Serum proteins, Proteomic profiling, Proximity extension assay, Machine learning

## Abstract

**Background:**

Immune checkpoint inhibitors (ICIs), such as anti-programmed cell death protein 1 (PD-1) and anti-cytotoxic T-lymphocyte associated protein 4 (CTLA-4), have transformed the management of stage III melanoma in the neoadjuvant setting. However, a substantial proportion of patients do not derive benefit from ICI therapy. To improve clinical outcomes, there remains a critical unmet need to identify early biomarkers of response to neoadjuvant immunotherapy in stage III melanoma.

**Methods:**

In this study, we performed longitudinal serum proteomic profiling in 39 patients undergoing neoadjuvant combination anti-PD-1 and anti-CTLA-4 therapy. Using a multiplex proximity extension assay, we measured 702 proteins at three timepoints: baseline, early on-treatment (3–4 weeks after treatment initiation), and pre-surgery (4–8 weeks post-treatment).

**Results:**

The most pronounced differences between major pathological responders (MPR) and non-MPR patients were detected at baseline and were linked to interferon gamma (IFNγ) signalling, but these differences diminish at later timepoints. A 10-protein IFNγ-associated signature derived from baseline serum profile achieved an AUC of 0.68 for predicting pathological response, comparable to a previously reported tumour-based IFNγ gene signature (AUC = 0.67).

**Conclusions:**

These findings support the use of circulating protein signatures as minimally invasive, scalable biomarkers to inform early treatment decisions in the neoadjuvant setting.

**Supplementary Information:**

The online version contains supplementary material available at 10.1186/s12935-025-04067-4.

## Background

Immune checkpoint inhibitors (ICIs), including anti-PD-1, anti-CTLA-4, and anti-lymphocyte activation gene 3 (LAG-3), have transformed the treatment of advanced melanoma [1–3]. The combination of nivolumab (anti-PD-1) plus ipilimumab (anti-CTLA-4) has achieved a 10-year overall survival (OS) rate of 43% and a melanoma-specific survival (MSS) rate of 52% for patients with advanced melanoma [2]. Similarly, the combination of nivolumab plus relatlimab (anti-LAG-3) demonstrated durable efficacy with a 5-year OS of 48.7% and MSS of 58.2% [3]. These ICIs have also shown clinical benefit in the neoadjuvant and adjuvant settings for patients with stage III or resectable stage IV melanoma, with neoadjuvant treatment significantly improving event-free survival compared to adjuvant therapy [4, 5]. Notably, neoadjuvant regimens combining anti-PD-1 with anti-CTLA-4 or anti-LAG-3 have achieved major pathological response (MPR; defined as ≤ 10% viable tumour cells in the index lymph node) rates of 59% (*n* = 212, Phase 3 trial) [4] and 63% (*n* = 30, Phase 2 trial) [6]. Although the 3-year recurrence-free survival (RFS) exceeds 90% in patients who achieve MPR, many patients without MPR also remain recurrence-free − 79% in patients with partial pathological response (pPR), and 41% in patients with pathological non-response (pNR) [7]. This underscores the need for better predictive biomarkers to stratify outcomes, particularly among non-responders.

Most predictive ICI biomarkers are derived from tumour biopsies and reflect features of tumour antigenicity and immune activity within the tumour microenvironment. High tumour mutation burden (TMB) and interferon gamma (IFNγ)-related gene expression are key predictors of ICI response. A multivariable model combining both features predicted response in patients with advanced melanoma, with an area under the curve (AUC) of 0.84, while the IFNγ gene signature alone had an AUC of 0.76 [8].

Many biomarker studies rely on tumour-derived genomic and transcriptomic data, which require suitable tissue specimens, costly sequencing and may not fully capture intra-patient tumour heterogeneity. In contrast, circulating biomarkers offer practical advantages, including ease of sampling, longitudinal monitoring, and the ability to capture systemic disease dynamics. A recent study using machine learning on plasma protein data outperformed other tissue-based biomarkers in predicting ICI response (AUC 0.54–0.80) in a cohort of 174 patients with metastatic melanoma [9]. However, the predictive potential of circulating protein biomarkers remains underexplored in stage III melanoma patients receiving neoadjuvant ICI.

In this study, we comprehensively profiled 702 proteins in serum samples from stage III melanoma patients (*n* = 39) treated with neoadjuvant anti-PD-1 and anti-CTLA-4 therapy to identify circulating ICI biomarkers. Protein expression was assessed at baseline, early during treatment, and prior to surgery. We report that baseline expression of a 10-protein IFNγ signature is associated with ICI response (AUC 0.68), highlighting its potential as a clinically useful, dynamic predictor of treatment response. These findings provide proof-of-concept that a circulating IFNγ-associated protein signature can stratify patients by pathological response in the neoadjuvant setting.

## Methods

### Patients

This study included 39 patients with resectable stage III nodal melanoma treated with neoadjuvant ipilimumab and nivolumab. Written informed consent was obtained from all participants. Biospecimen samples were acquired with consent from the Melanoma Institute Australia (MIA) Biospecimen Tissue Bank. Ethics approval for sample collection and use was obtained from the Human Research Ethics Committee at the Royal Prince Alfred Hospital (X15-0454 & 2019/ETH06874). All procedures were conducted in accordance with institutional guidelines, national regulations, and the Declaration of Helsinki.

Of the 39 patients, 25 received two cycles of 1 mg/kg ipilimumab plus 3 mg/kg nivolumab, while 14 were treated with two cycles of 3 mg/kg ipilimumab plus 1 mg/kg nivolumab every three weeks [10, 11]. Pathological response was assessed according to The International Neoadjuvant Melanoma Consortium (INMC) criteria [12] and patients were categorized as pathological complete response (pCR; 0% viable tumour cells), near-complete response (near pCR, 1 to ≤ 10% viable tumour cells), partial response (pPR; >10 to ≤ 50% viable tumour cells), or non-response (pNR; >50% viable tumour cells). Clinical variables including age, sex, AJCC (American Joint Committee on Cancer, 8th edition) staging, and baseline lactate dehydrogenase (LDH) levels were collected (Table 1).

**Table 1:** Patient characteristics and treatment outcomes

Characteristics	Patients (*n* = 39)
Age in years*,** median (range)**	62 (19–85)
**Sex**,** n (%)**	
Male	25 (64)
Female	14 (36)
**Disease Stage (AJCC 8th edition)**,** n (%)**	
IIIB	21 (54)
IIIC	18 (46)
**LDH**,** n (%)**	
Normal	36 (92)
Elevated	3 (8)
**Treatment, n (%)**	
Ipilimumab 3 mg/kg + Nivolumab 1 mg/kg	14 (36)
Ipilimumab 1 mg/kg + Nivolumab 3 mg/kg	25 (64)
**Best RECIST Response**,** n (%)**	
CR	4 (10)
PR	18 (46)
SD	14 (36)
PD	2 (5)
N/E	1 (3)
**Pathological Response**,** n (%)**	
Major Pathological Response (MPR)	27 (69)
pCR	17 (43)
Near pCR	10 (26)
Non-Major Pathological Response (non-MPR)	12 (31)
pPR	3 (8)
pNR	9 (23)

*Age at start of treatment

AJCC, American Joint Committee on Cancer; LDH, lactate dehydrogenase; RECIST, Response evaluation criteria in solid tumours; CR, complete response; PR, partial response; SD, stable disease; PD, progressive disease; N/E, not evaluated; pCR, pathological complete response; pPR, pathological partial response; pNR, pathological non-response

### Patient serum samples

Peripheral blood was collected from patients prior to ICI therapy (baseline, 0–28 days prior to treatment), early during treatment (EDT, 3–4 weeks after treatment initiation), and prior to surgery (pre-surgery, 4–8 weeks after treatment). Blood was drawn into VACUETTE^®^ CAT serum clot activator tubes (Cat no. 455092, Greiner Bio-One), allowed to clot at room temperature for 30 min, and centrifuged at 1300–2000 x g for 10–15 min. The resulting serum was separated and stored at −80 °C until analysis.

### Serum proteomic immunoprofiling

Proteomic profiling was conducted on 104 matched serum samples (baseline, *n* = 39, EDT, *n* = 26, and pre-surgery, *n* = 39) using a high-throughput, affinity-based proximity extension assay (Olink^®^). Target protein is recognized by a matched pair of oligonucleotide-labelled antibodies, which generates a unique DNA sequence via a proximity-dependent DNA polymerization event. This resulting sequence is amplified and quantified using next-generation sequencing.

Protein expression was measured using two predefined 384-plex panels (Explore 384 Inflammation and Oncoy) detecting 733 proteins. All samples were randomized across assay plates and processed in accordance with Olink’s standard workflow, including normalization using built-in internal controls. Protein abundance was reported as Normalized Protein expression (NPX), presented on a log scale.

Of the 733 proteins, 17 proteins (GBP2, BCL2L11, BID, LTA, PTPRM, RAB6A, CD40LG, IDS, CLEC7A, HGF, MGLL, UXS1, BIRC2, MED18, MPI, KIFBP, and ANGPTL7) did not meet Olink’s quality control criteria and were excluded. Another 14 proteins (IL24, CEP164, RGS8, CXCL14, NFATC3, IL1A, VASH1, HLA-E, MAGED1, CCT5, RANGAP1, FES, NUDT2, ATP6V1D) were excluded due to NPX values below the lower limit of detection in more than 75% of samples, with 702 proteins retained for further analysis.

### Normalization of NPX value

We applied RUVg (Remove Unwanted Variation using control genes) normalization to mitigate unwanted technical noise [13]. RUVg was performed using the RUVSeq package (version 1.40.0), with the number of unwanted factors set to k = 3. Negative control proteins were selected from a reference list of housekeeping genes derived from bulk RNA-seq data (bulkRNAseqHK) [14]. Proteins corresponding to these genes were identified using the *segList_ensemblGeneID* function from the scMerge R package (version 1.22.0) [14]. Of the 96 proteins in our dataset, 12 with variance >2 were excluded, resulting in 84 housekeeping proteins used as negative controls.

### Differential protein expression analysis

Differential expression analysis was performed separately at each timepoint to compare differences between pathological response using the limma R package (version 3.62.2). As principal component analysis revealed no evident clustering by clinical variables, no additional covariates were included in the design matrix. A *P*-value < 0.05 is considered significant.

### Pathway activity estimation and differential analysis

Single-sample gene set enrichment analysis (ssGSEA) was conducted using the GSVA R package (version 1.40.1) with the 50 Hallmark gene sets from the Molecular Signatures Database (MSigDB) [15]. Limma (version 3.62.2) was used to assess differential pathway activity between pathological response at each timepoint. A *P*-value < 0.05 is considered significant.

### Upstream regulator analysis for differentially expressed proteins

Differentially expressed proteins (*P*-value < 0.05) at baseline were imported into Ingenuity Pathway Analysis (IPA) (QIAGEN Inc., https://digitalinsights.qiagen.com/IPA) for upstream regulator analysis. An activation z-score is computed to predict whether each regulator is likely to be activated or inhibited [16]. A positive z-score indicates likely activation, a negative z-score suggests inhibition, and the z-score magnitude reflects the confidence in the prediction. *P*-value indicates the statistical significance of overlap between observed and predicted downstream targets, calculated using Fisher’s exact test.

### IFNγ-associated protein signature to predict pathological response

We estimated the predictive performance of IFNγ‑related proteins by building a classification model using Generalized Linear Model (GLM) to discriminate pathological response in the ClassifyR package [17]. Features were selected within each cross‑validation fold using a t‑test, with parameters *selectionMethod* = “auto” and *nFeatures* = c(10, 15, 20). This analysis was conducted independently at each timepoint (baseline, EDT, and pre‑surgery). For sensitivity analysis, we repeated the prediction with three classification algorithms including Random Forest (RF), Support Vector Machine (SVM), and Diagonal Linear Discriminant Analysis (DLDA). We constructed three classification models using the ClassifyR framework: a clinical model using three clinical variables (age, sex, and stage), a protein-based model with the 188 IFNγ-associated proteins, and a combined model integrating both the clinical and protein features. All models were built using the GLM method. The confidence intervals (CI) of the area under the curve (AUC) were estimated using the *ci.auc* function from the pROC package (v1.18.5) via bootstrapping, with CI derived from the empirical quantiles of the bootstrapped AUC distributions. Sensitivity, specificity, positive predictive value (PPV) and negative predictive value (NPV) were calculated, and their CI were estimated using the *BinomCI* function from the DescTools package with the Wilson method.

## Results

### Patient and sample characteristics

**Fig. 1:**
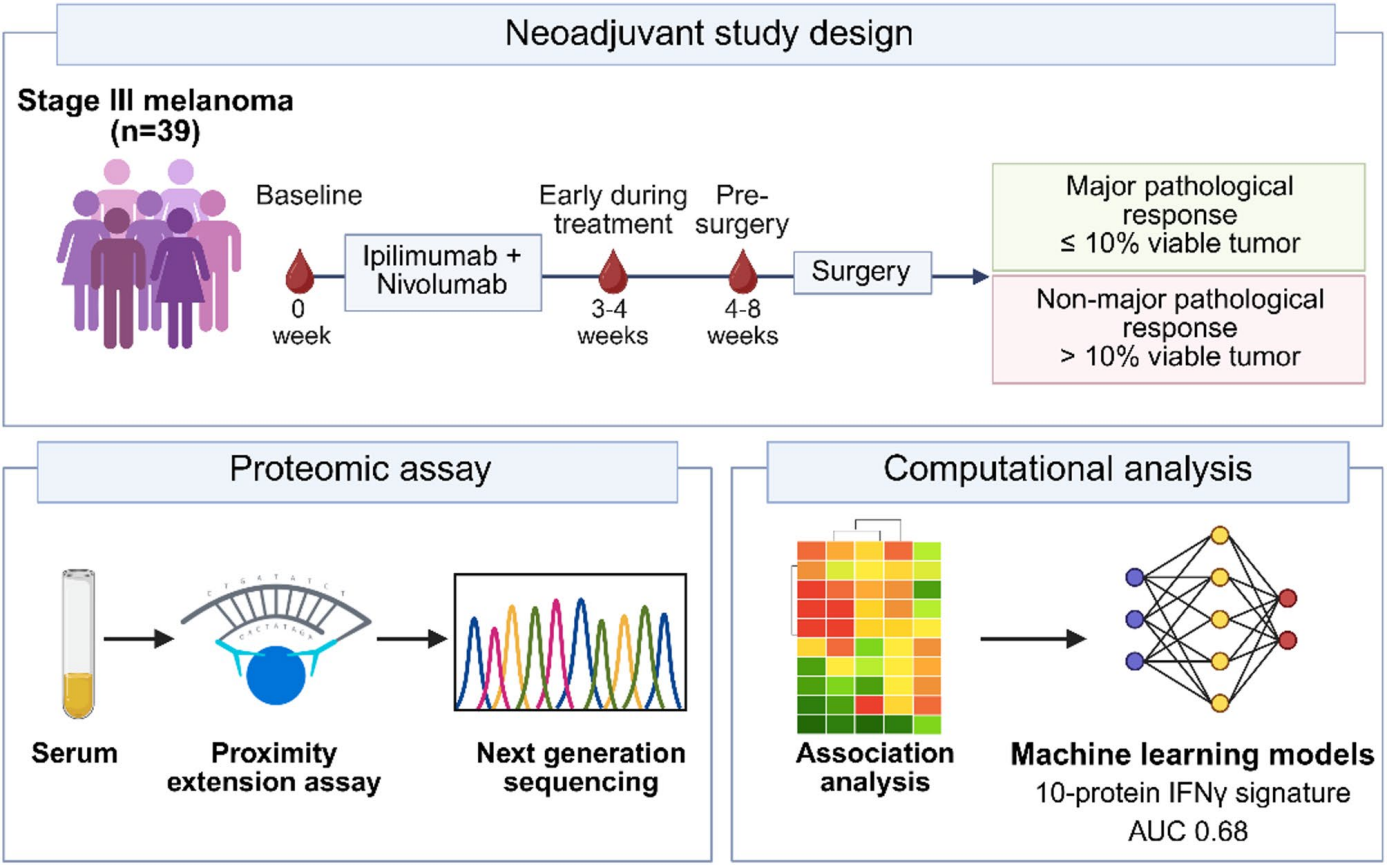
Schematic of the study design. Serum samples were collected from 39 stage III melanoma patients at baseline (0–28 days prior to treatment), early during treatment (EDT, 3–4 weeks after treatment) and pre-surgery (4–8 weeks after treatment) following neoadjuvant anti-PD-1 and anti-CTLA-4 therapy. Pathological response was evaluated on the resection specimen and defined as major pathological response (MPR, ≤ 10% viable tumour cells) or non-major pathological response (non-MPR, > 10% viable tumour cells). 702 proteins in serum samples were analysed using the Olink platform. Machine learning models were utilised to assess the predictive performance of circulating proteins in differentiating patients with MPR from non-MPR

This study included 104 serum samples from 39 patients with resectable stage IIIB-C nodal melanoma receiving neoadjuvant anti-CTLA-4 (ipilimumab) and anti-PD-1 (nivolumab). All patient samples were obtained from Melanoma Institute Australia. Patient matched serum samples were collected at baseline (0–28 days prior to treatment initiation, *n* = 39), early during treatment (EDT; 3–4 weeks after treatment initiation, *n* = 26), and pre-surgery (4–8 weeks after treatment initiation, *n* = 39) (Fig. 1). Patients received two cycles of either ipilimumab (1 mg/kg) plus nivolumab (3 mg/kg) (25/39; 64%) or ipilimumab (3 mg/kg) plus nivolumab (1 mg/kg) (14/39; 36%) every 3 weeks. The median age of the patient cohort was 62 years (range 19–85), and 64% (25/39) were male. Most patients had normal LDH levels (36/39; 92%) and stage IIIB disease (21/39; 54%). Pathological response was evaluated on the resection specimen and defined as pathological complete response (pCR; 0% viable tumour cells), near pCR (1 to ≤ 10% viable tumour cells), pathological partial response (pPR; >10 to ≤ 50% viable tumour cells) and pathological non-response (pNR; >50% viable tumour cells) [12]. Major pathological response (MPR) was defined as patients with pCR or near pCR, as both represent minimal residual viable tumour and are strongly associated with favourable clinical outcomes [18]. Using this definition, 27/39 (69%) patients achieved MPR while the remaining 12/39 (31%) patients were classified as non-MPR, including those with pPR (3/39) or pNR (9/39). Patient demographics and clinical characteristics are summarized in Table 1.

### Differentially expressed proteins between responding and nonresponding patients

**Fig. 2:**
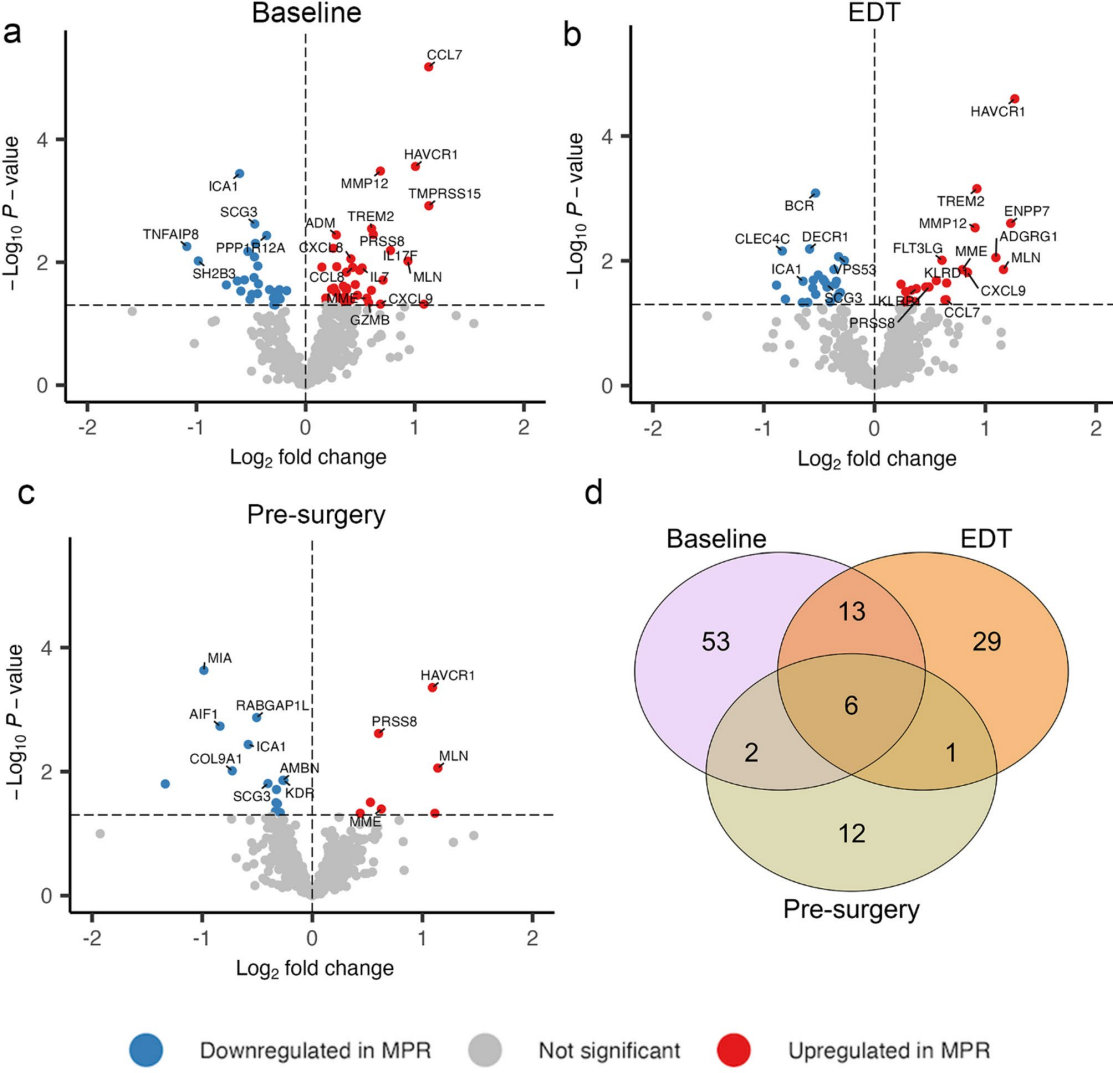
Differential expression analyses of 702 proteins reveal more significantly altered proteins at baseline than at EDT or pre-surgery. Volcano plots showing significantly differentially expressed proteins (*P*-value < 0.05) between MPR and non-MPR at (**a**) baseline, **(b**) EDT, and (**c**) pre-surgery. Proteins upregulated in MPR patients shown in red and downregulated proteins in blue. (**d**) Venn diagram shows the overlap of differentially expressed proteins across the three timepoints. Log₂ fold change values represent the difference in expression between MPR versus non-MPR

Expression of 702 serum proteins was quantified using the highly multiplexed proximity extension assay (PEA; Olink Proteomics, Uppsala, Sweden) (Fig. 1). We first examined the temporal dynamics of protein expression in response to ICI treatment by comparing responding (MPR) and non-responding (non-MPR) patients at baseline, EDT and pre-surgery using differential expression analysis. While only one protein met the stringent FDR < 0.05 threshold at baseline, for the purpose of this discovery study, we also considered proteins with nominal P-values < 0.05 to capture broader biological signals, with the understanding that this may include an increased proportion of false positive. Volcano plots of the differentially expressed serum proteins between MPR vs. non-MPR (Fig. 2a-c) reveal a greater number of significantly altered proteins (*P*-value < 0.05) at baseline (*n* = 74) compared to EDT (*n* = 49) and pre-surgery (*n* = 21) (Table S1). At baseline, expression of pro-inflammatory cytokines, chemokines (e.g. CCL7, CXCL8, CCL8, CXCL9) and immune modulatory markers associated with T cell activity (e.g. IL7, IL17F, HAVCR1, GZMB) were upregulated in MPR. In contrast, non-MPR patients showed upregulated expression of immune-suppressive molecules, including TNFAIP8 and SH2B3 (Fig. 2a).

At EDT, several pro-inflammatory cytokines and immune modulatory markers (CXCL9, CCL7, HAVCR1) remain elevated in MPR patients (Fig. 2b). In addition, proteins associated with natural killer (NK) and T cell activity, such as KLRD1 and KLRB1, were upregulated in MPR patients at this timepoint. At the pre-surgery timepoint, HAVCR1 was the only baseline-elevated protein that remained upregulated in MPR patients. In contrast, the expression of several other proteins, including MIA, AIF1, KDR was significantly reduced compared to non-MPR patients (Fig. 2c). Consistent with these findings, only six proteins were differentially expressed between MPR and non-MPR patients across all three timepoints – four upregulated (HAVCR1, MLN, PRSS8, MME) and two downregulated (ICA1, SCG3) (Fig. 2d).

### Differential pathway alterations in responding and nonresponding patients

Next, we examined changes in biologically relevant pathways, with a focus on those differentially altered between MPR and non-MPR patients across the three timepoints. To this end, we applied single-sample gene set enrichment analysis (ssGSEA) across the 50 Hallmark pathways [15] to identify pathway-level alterations. This approach also revealed more pronounced differences in pathways at baseline compared to EDT and pre-surgery between the two response groups. In particular, IL6_JAK_STAT3 signalling was enriched in MPR patients, while the WNT_BETA_CATENIN signalling was enriched in non-MPR patients at baseline, but not at later timepoints (Fig. S1). Overall, these findings indicate that differences in immune-related pathway activity are more prominent in the serum of patients prior to ICI initiation, but this distinction was less striking following treatment onset.

**Fig. 3:**
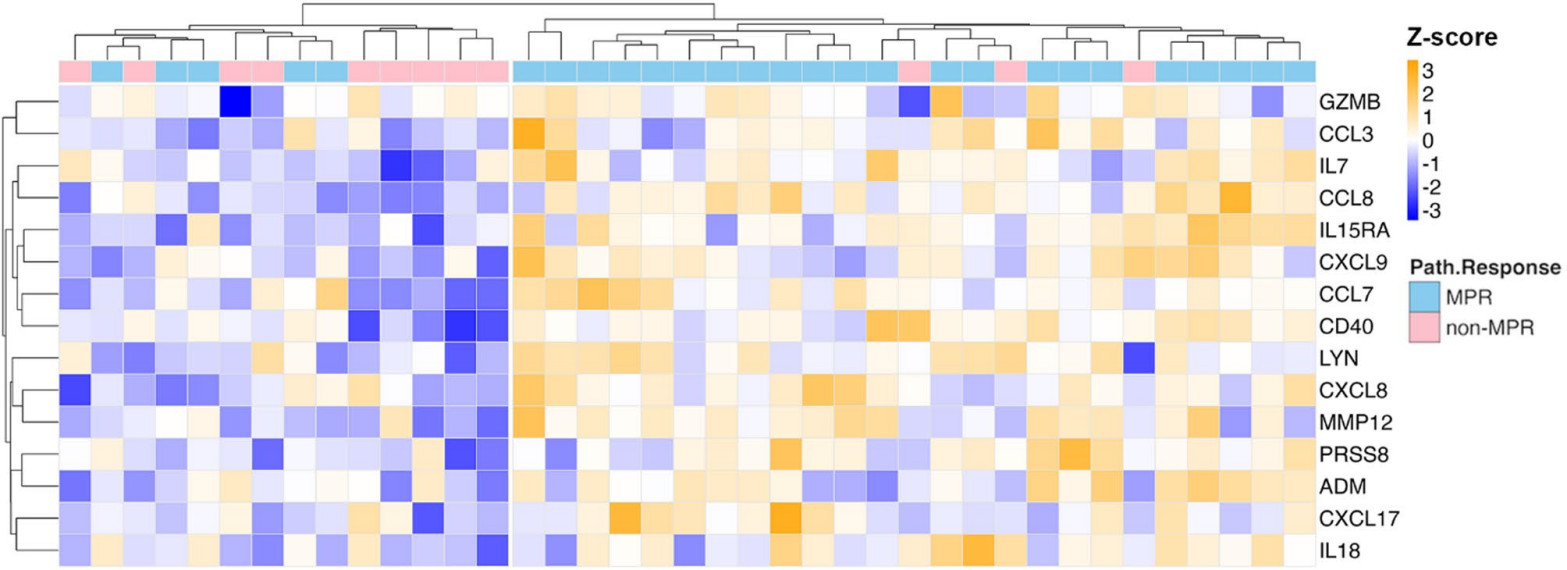
IFNγ-associated differentially expressed proteins. Heatmap showing the expression of 15 proteins differentially expressed (*P*-value < 0.05) between MPR and non-MPR patients at baseline. These 15 proteins are predicted downstream targets of IFNγ signalling based on the IPA analysis

Given the differences in enrichment of immune-related proteins and pathways between MPR and non-MPR patients at baseline, we further explored the relationship between the differentially expressed proteins and immune-related pathway activities. We performed a pathway enrichment calculation using Ingenuity Pathway Analysis (IPA) with an input of the 74 differential expressed proteins (*P*-value < 0.05) between MPR and non-MPR patients at baseline. Fifty upstream regulators were estimated to be highly activated in MPR patients compared to non-MPR patients (activation z-score > 2, *P*-value < 0.05) (Table S2). Of these, IFNγ had an activation z-score of 2.79 with 21 predicted downstream targets shown in Fig. S2. The baseline expression of the 15 differentially expressed (*P*-value < 0.05) downstream IFNγ targets show consistent upregulation in MPR compared to non-MPR patients (Fig. 3), indicating that IFNγ effectors may function as predictive biomarkers to stratify patient response prior to neoadjuvant ICI.

### Predictive performance of IFNγ-associated signature

**Fig. 4:**
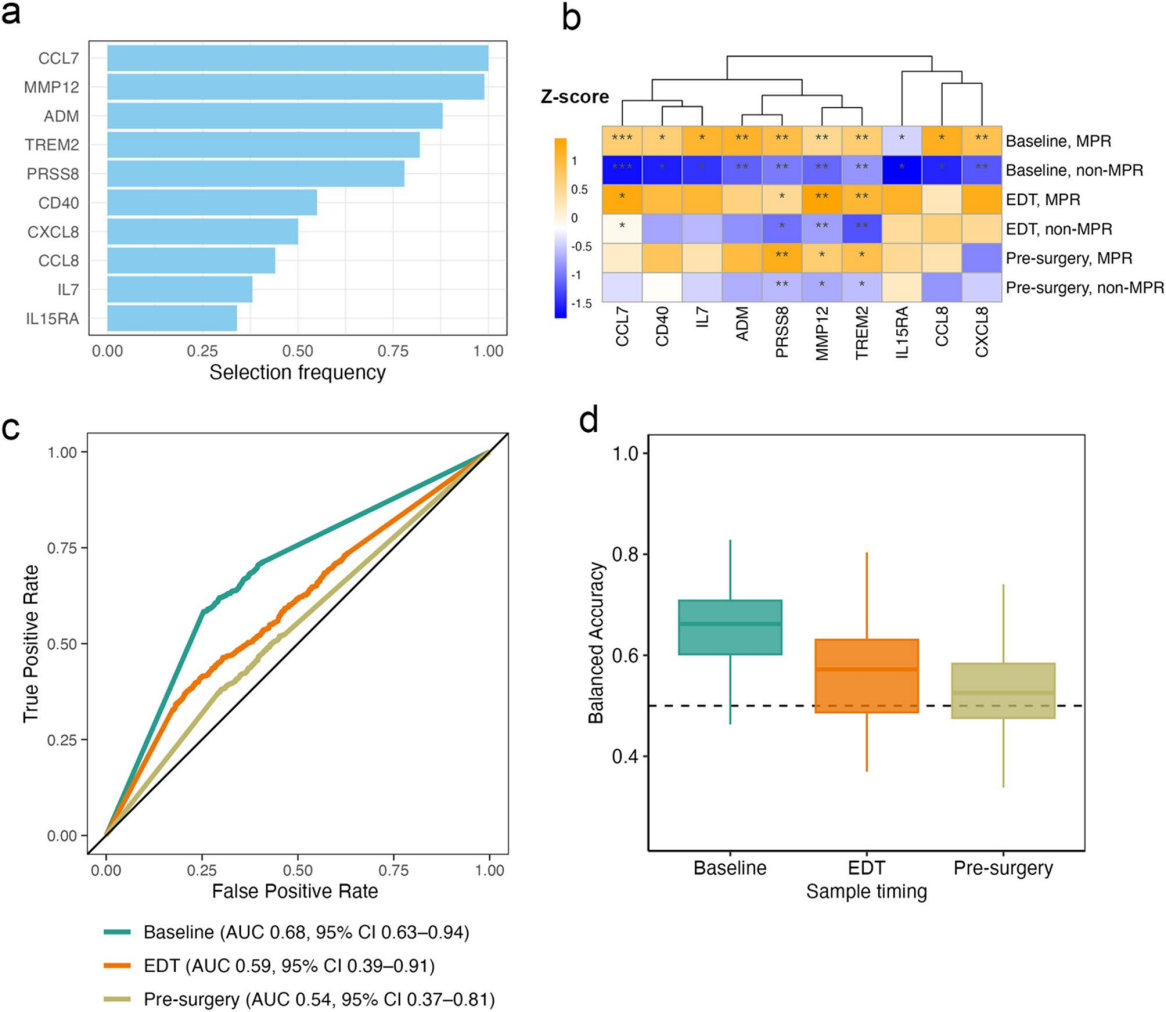
Predictive performance of IFNγ signatures. (**a**) Selection frequency of the 10 proteins identified from 5-fold cross-validation using GLM at baseline. These proteins comprise the baseline 10-protein IFNγ signature. (**b**) Expression of the 10 proteins in MPR and non-MPR patients at baseline, EDT and pre-surgery. Statistical significance assessed using Wilcoxon rank-sum test: ***P-value < 0.001, **P-value < 0.01, *P-value < 0.05. (**c**) Receiver operating characteristic curves for the three GLM models following feature selection on 188 IFNγ-associated proteins and model building at baseline, EDT and pre-surgery separately. (**d**) The corresponding balanced accuracy plot for the models described in (**c**)

Considering the relationship between serum IFNγ effectors and ICI response, we expanded our analysis to include 188 IFNγ-associated proteins; these proteins were selected from the union of MSigDB HALLMARK_INTERFERON_GAMMA_RESPONSE and IPA IFNγ downstream targets (Table S3). We performed feature selection on the 188 proteins using 5-fold cross validation repeated 100 times with a generalised linear model (GLM), yielding a 10-protein IFNγ signature at baseline (Table S4). Figure 4a shows the frequency with which each feature was selected across the 100 runs. The heatmap shows expression of all 10 proteins at the three timepoints: baseline, EDT, and pre-surgery, in MPR and non-MPR patients (Fig. 4b). The expression differences for each of these IFNγ target protein between responding and non-responding patients are shown in Fig. S3. Compared to baseline, eight of the ten IFNγ target proteins showed reduced differential expression between MPR and non-MPR patients at both EDT and pre-surgery. As a result, the discriminative power of this 10-protein IFNγ signature was not expected to persist at later timepoints.

At baseline, the 10-protein IFNγ signature achieved an AUC of 0.68 using the GLM, with additional classification performance metrics shown in Table S5. Applying the same approach with feature selection on the 188 IFNγ-associated proteins and model building for the other two timepoints, we observed an AUC of 0.59 at EDT and 0.54 at pre-surgery (Fig. 4c, d). This suggests that there is no alternative subset of IFNγ-associated proteins with discriminative potential at later timepoints, and IFNγ-associated signatures in the circulation may have little prognostic value once ICI treatment is initiated.

Additional sensitivity analysis showed that different modelling approaches (e.g. Diagonal Linear Discriminant Analysis (DLDA), Random Forest (RF) and Support Vector Machine (SVM)) using the same 188 IFNγ-associated proteins (Fig. S4) or using all 702 proteins from the Olink panel (Fig. S5) produced comparable results, with predictive performance declining at later timepoints. Incorporating clinical variables such as age, sex, and stage in the prediction modelling at baseline did not improve the predictive performance (Fig. S6), suggesting that the 10-protein IFNγ signature alone provides sufficient discriminative power.

## Discussion

This study presents a comprehensive longitudinal analysis of 702 circulating proteins in 39 stage III melanoma patients undergoing neoadjuvant immunotherapy. Protein- and pathway-level analyses revealed greater differences between MPR and non-MPR patients at baseline compared to later timepoints. Baseline protein differences were strongly associated with IFNγ-related activity, and a 10-protein IFNγ signature achieved an AUC of 0.68 for predicting pathological response. Importantly, the serum-derived IFNγ signature for identifying responders to neoadjuvant immunotherapy demonstrated predictive performance comparable to a previously reported tumour-based IFNγ gene signature (AUC = 0.67) [10]. These findings highlight the clinical potential of circulating IFNγ-related protein signatures as minimally invasive, scalable biomarkers for predicting immunotherapy response, and support their use as an orthogonal approach to guide patient stratification and treatment decision in the neoadjuvant setting.

Our longitudinal proteomic analyses revealed that the baseline differences in circulating proteins diminished following treatment initiation. While 74 proteins were differentially expressed at baseline, only six remained significantly altered between MPR and non-MPR patients across all three timepoints. Of these, four proteins consistently upregulated in MPR patients (HAVCR1, MLN, PRSS8, MME) are associated with immune activation and inflammation [19–22], whereas the two proteins upregulated in non-MPR patients (ICA1, SCG3) are linked to vesicle trafficking and secretory pathways [23, 24]. The reduction in discriminatory signal after treatment is likely attributable to the pharmacodynamic effects of ICIs, which induce broad systemic and local immune activation, including increased IFNγ signalling, regardless of treatment response [25]. As such, therapy-induced upregulation of IFNγ-regulated cytokines and inflammatory mediators in the circulation may obscure their predictive value during therapy. Consistent with this, the predictive performance of the serum IFNγ signature was highest at baseline and declined at subsequent timepoints after treatment initiation. In contrast, tumour-derived IFNγ transcriptomic signatures demonstrate superior predictive performance in on-treatment samples [26], with greater induction of IFNγ response genes, particularly antigen presentation genes, in responders following ICI therapy [25]. Moreover, melanoma patients with high early on-treatment tumour IFNγ scores despite low baseline values still achieved pathological responses after neoadjuvant ICI [11]. This divergence in the dynamics of circulating vs. tumour IFNγ signatures likely reflects therapy-induced T cell infiltration and localised immune remodelling in the tumour microenvironment – features not captured in the circulation [25, 27].

Previous studies have established that tumour-derived IFNγ-related gene expression signatures can predict immunotherapy outcomes, highlighting this pathway as a key correlate of response. For instance, a 10-gene IFNγ signature was predictive of response to the PD-1 inhibitor pembrolizumab in patients with advanced melanoma [28]. In patients with stage III melanoma receiving neoadjuvant therapy, baseline intratumoral IFNγ scores also predict responses, with 95% of patients with IFNγ-high tumours achieving a pathological response, compared to 59% in patients with IFNγ-low tumours [11]. The predictive performance of the baseline IFNγ-associated gene signature for differentiating ICI response achieved an AUC of 0.69 in a mixed cohort of stage III melanoma patients with and without adjuvant therapy [29] and an AUC of 0.67 in stage III melanoma patients receiving neoadjuvant ICI therapy [10]. However, the detection and relevance of IFNγ activity in circulation remains unexplored. Our study provides critical evidence that serum proteins associated with IFNγ signalling can differentiate MPR from non-MPR at baseline. Specifically, we identified a 10-protein IFNγ signature derived from the canonical Hallmark IFNγ pathway and IPA dataset; these proteins are key modulators of immune cell recruitment and activation (e.g. CCL3, CCL4, CCL7, CD40) [30–33], inflammation (MMP12, PTX3) [34, 35] and immune tolerance (LAG3, IL11) [36, 37], underscoring the role of IFNγ in mediating anti-tumour immunity. Additionally, feature selection on the full Olink protein panel yielded a 10-protein signature that included seven non-IFNγ-associated proteins (HAVCR1, ICA1, AMBP, TMPRSS15, SCG3, PPP1R12A and LRP1) that were amongst the top differentially expressed proteins between MPR and non-MPR patients at baseline; these could additionally serve as potential biomarkers of immunotherapy response. Taken together, our findings demonstrate that circulating proteins can serve as effective biomarkers for predicting treatment response, complementing conventional tumour biopsy-dependent prediction approaches.

Although the sample size is limited (*n* = 39), with EDT samples available for only 26 patients, to our knowledge, this represents the first proof-of-concept demonstration that a circulating IFNγ signature can differentiate response to neoadjuvant ICI. Importantly, the limited availability of EDT samples is unlikely to affect the overall conclusions, as the most pronounced differences between MPR and non-MPR patients were detected at baseline. The limited sample size also restricts statistical power, and we included proteins with nominal P-values < 0.05 to capture broader biological signals, acknowledging the increased risk of false positives. While we do not have an external dataset for independent validation, we have conducted extensive internal validation, including repeated cross-validation, sensitivity analyses, and model perturbation tests. The consistent performance observed across four distinct machine learning models underscores the robustness and reliability of our findings.

## Conclusions

We demonstrate that longitudinal proteomic profiling offers an orthogonal approach to interrogate and predict treatment response in stage III melanoma patients receiving neoadjuvant immunotherapy. The findings provide proof-of-concept that circulating proteins, particularly those associated with IFNγ signalling, can function as early, non-invasive predictors of pathological response. Pending validation in larger and more diverse cohorts, these circulating protein markers may serve as clinically useful tools to guide early treatment decisions in stage III melanoma.

## Supplementary Information

Supplementary Material 1.

## Data Availability

The data that support the findings of this study are provided in the supplementary information files. All additional datasets are available from the corresponding author upon reasonable request. The code used in the analysis is provided as part of a git repository and can be accessed from: https://github.com/SydneyBioX/Melanoma_neoadjuvant_Olink.

## Abbreviations

AJCC: American Joint Committee on Cancer
AUC: Area under curve
CTLA-4: Cytotoxic T-lymphocyte associated protein 4
CI: Confidence interval
CR: Complete response
DLDA: Diagonal linear discriminant analysis
EDT: Early during treatment
GLM: Generalized linear model
ICIs: Immune checkpoint inhibitors
IFNγ: Interferon gamma
INMC: International Neoadjuvant Melanoma Consortium
IPA: Ingenuity Pathway Analysis
LAG-3: Lymphocyte activation gene 3
LDH: Lactate dehydrogenase
PD-1: Programmed cell death 1
MPR: Major pathological response
MSigDB: Molecular Signatures Database
MSS: Melanoma-specific survival
NGS: Next-generation sequencing
NPX: Normalized protein expression
OS: Overall survival
PEA: Proximity extension assay
pCR: Pathological complete response
PD: Progressive disease
pNR: Pathological non-response
pPR: Pathological partial response
PR: Partial response
RECIST: Response evaluation criteria in solid tumours
RFS: Recurrence-free survival
RF: Random forest
ROC: Receiver operating characteristic
RUVg: Remove unwanted variation using control genes
SD: Stable disease
ssGSEA: Single-sample gene set enrichment analysis
SVM: Support vector machine
TMB: Tumour mutation burden
