# Supplementary material for: Circulating IFNγ-associated protein signatures predict response to neoadjuvant immunotherapy in patients with stage III melanoma
Source: Cancer Cell Int. 2025 Nov 25;25:424. doi: 10.1186/s12935-025-04067-4 (PMC12648901; doi:10.1186/s12935-025-04067-4)
Supplement: Supplementary file 1 — Supplementary Material 1. [file 12935_2025_4067_MOESM1_ESM.pdf]

Supplementary Figures

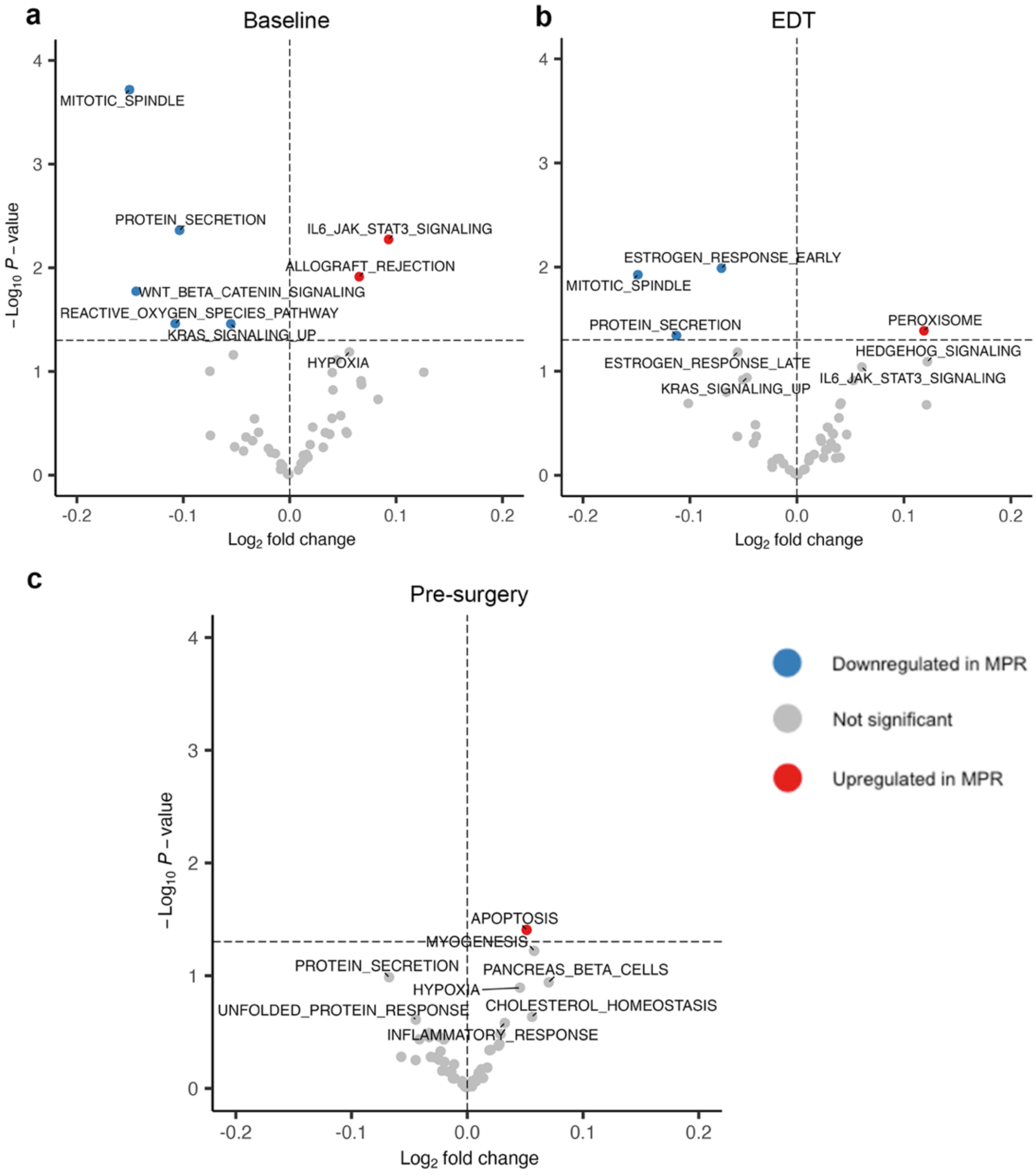

Figure S1. Differential expression analysis of ssGSEA Hallmark pathway scores

Volcano plots showing significantly differentially expressed (*P*-value < 0.05) Hallmark pathways scored by single-sample gene set enrichment analysis (ssGSEA) between MPR and non-MPR patients at (a) baseline, (b) EDT, and (c) pre-surgery. Pathways upregulated in MPR patients shown in red and downregulated pathways shown in blue. Log_2 fold change values represent the difference in ssGSEA score between MPR and non-MPR groups.

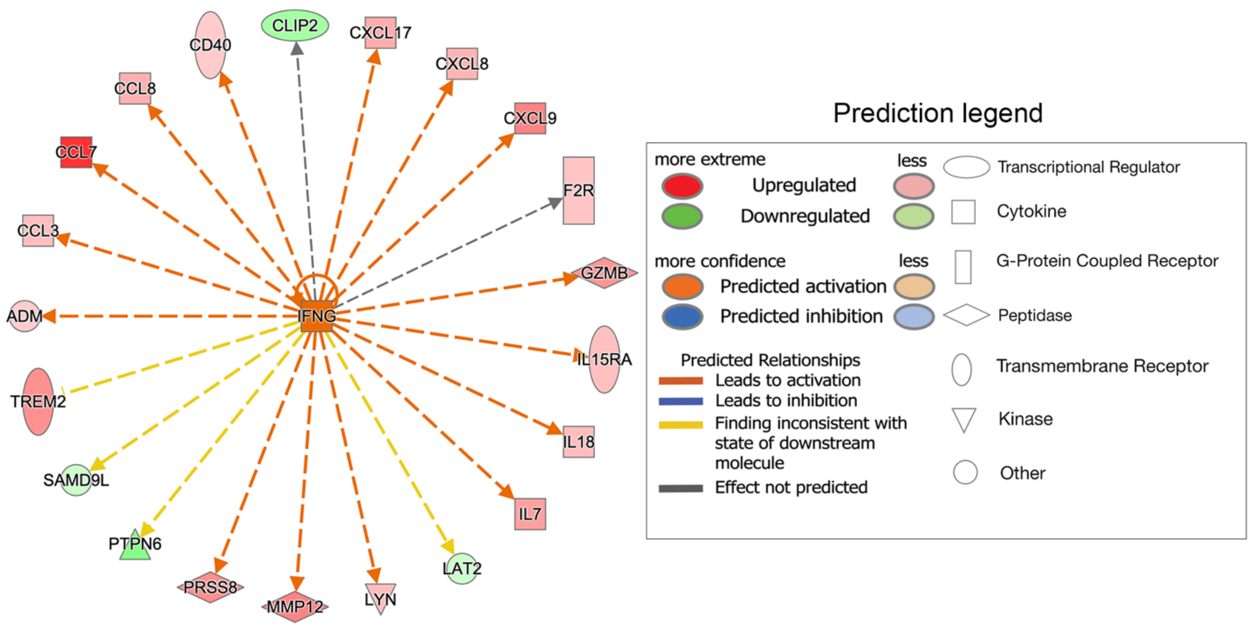

Figure S2. Predicted IFNγ signalling effector proteins identified from Ingenuity Pathway Analysis (IPA) upstream regulator analysis

Network graph showing IFNγ as an upstream regulator of the 21 downstream effector proteins that were differentially expressed (*P*-value < 0.05) between MPR and non-MPR patients at baseline.

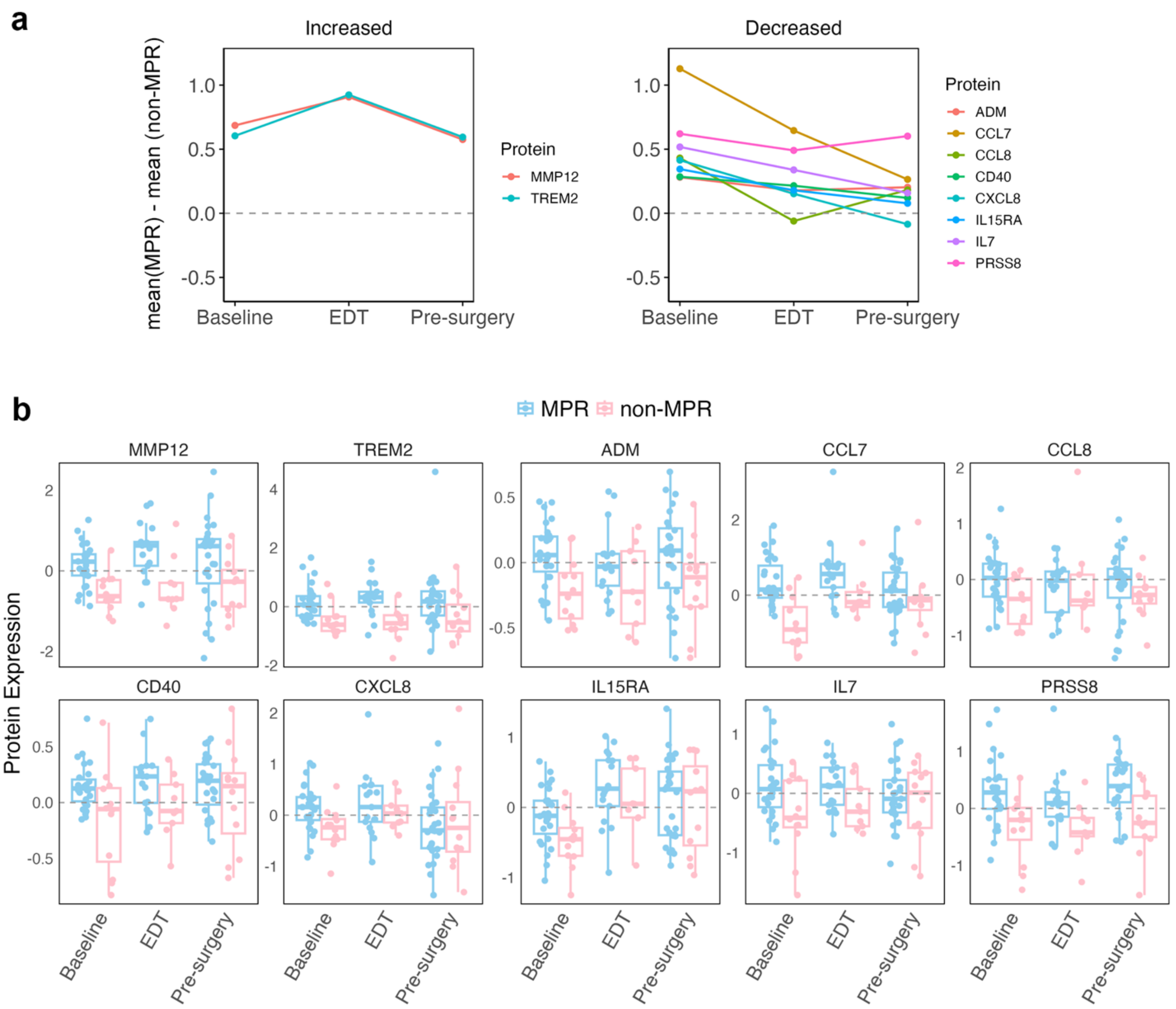

Figure S3. Expression dynamics of the proteins in the baseline 10-protein IFNγ signature across treatment timepoints

(a) Line plots showing differences in expression of the 10 IFNγ proteins between MPR and non-MPR patients at baseline, EDT, and pre-surgery. (b) Boxplots showing expression of the 10 IFNγ proteins in MPR (blue) and non-MPR (pink) patients across three timepoints, with each dot representing an individual patient.

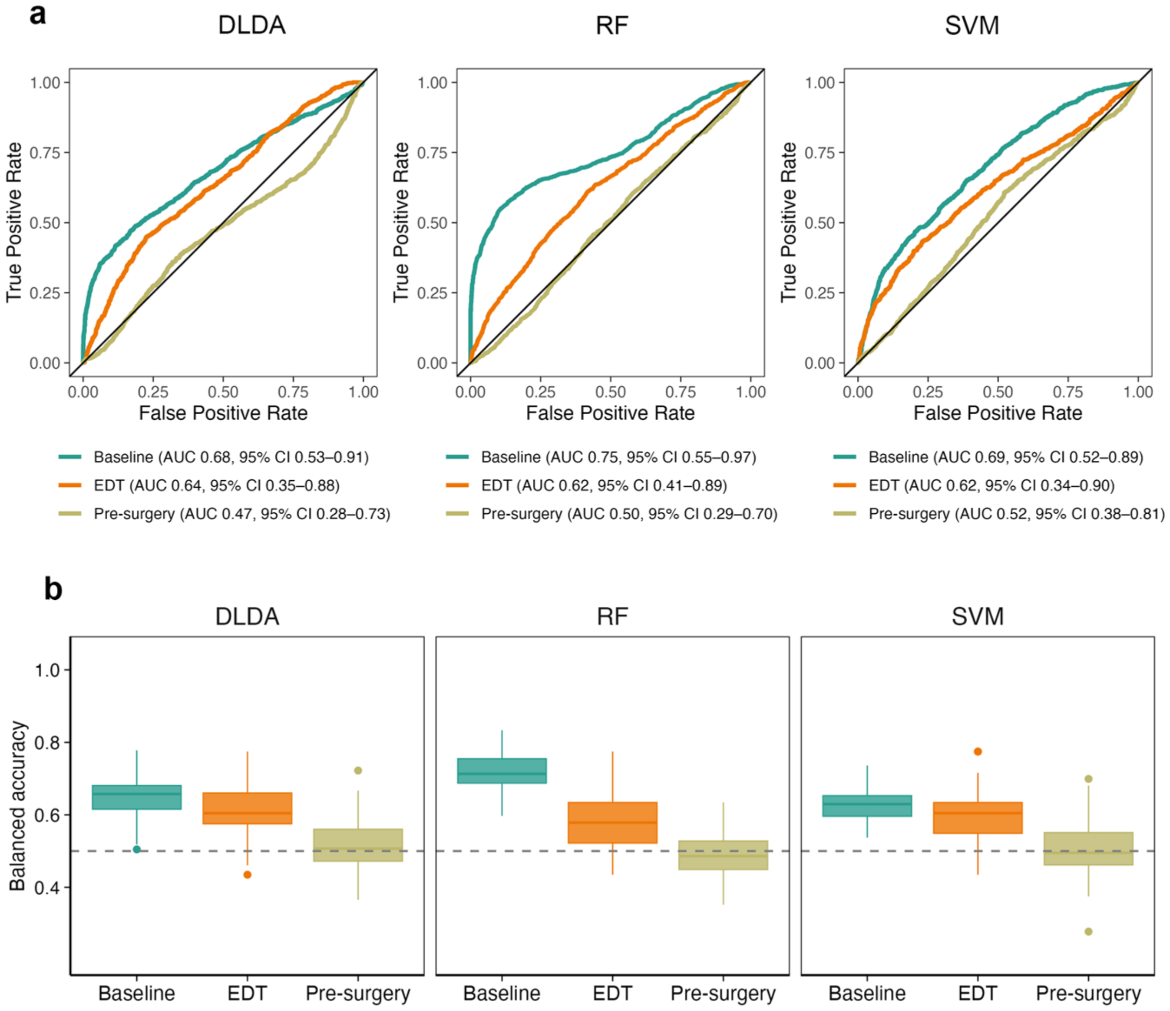

Figure S4. Predictive performance of alternative machine learning models using the 188 IFNγ-associated proteins at baseline

(a) Receiver operating characteristic curves and (b) balanced accuracy plots showing the predictive performance of the 188 IFNγ-associated proteins using each model (Diagonal Linear Discriminant Analysis (DLDA), Random Forest (RF) and Support Vector Machine (SVM)) from 5-fold cross-validation repeated 100 times. This analysis was conducted independently at each timepoint (baseline, EDT, pre-surgery).

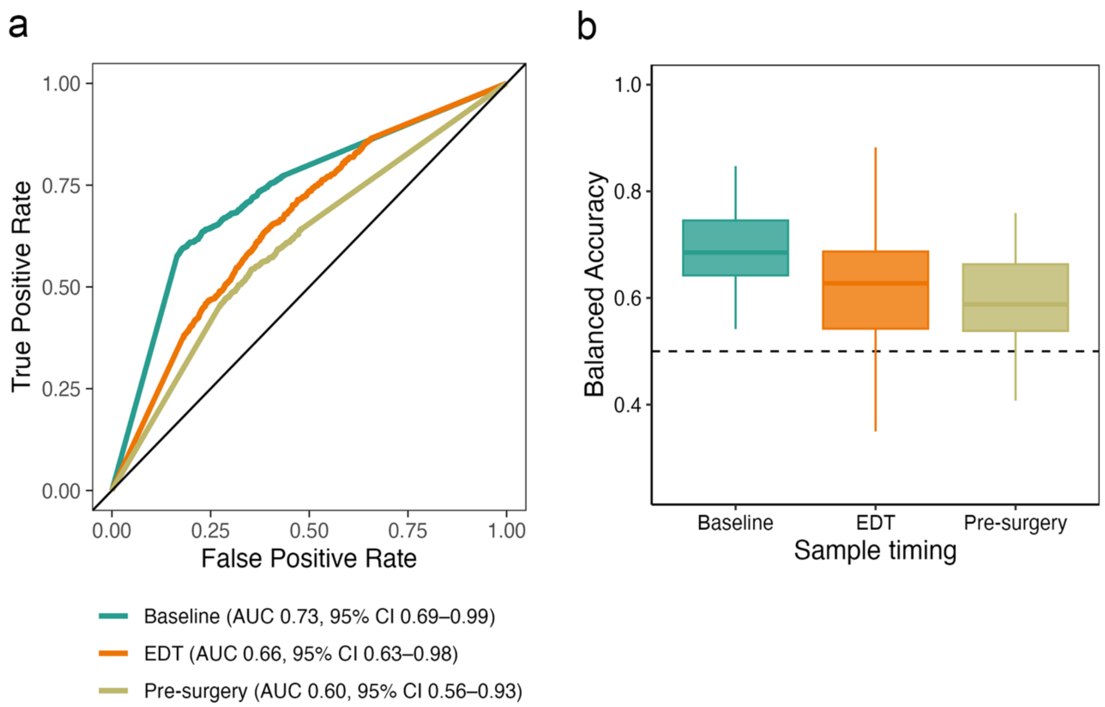

Figure S5. Predictive performance of GLM using all 702 circulating proteins.

(a) Receiver operating characteristic curves and (b) balanced accuracy plot showing the predictive performance of the GLM using all 702 circulating proteins with 5-fold cross-validation repeated 100 times. This analysis was conducted independently at each timepoint (baseline, EDT, pre-surgery). The alternative 10-protein signature derived at baseline based on 702 circulating proteins includes three IFNγ-associated proteins (CCL7, MMP12, and ADM) which overlap with the 10-protein IFNγ signature identified from feature selection on the 188 IFNγ-associated proteins at baseline. The remaining seven non-IFNγ-associated proteins (HAVCR1, ICA1, AMBP, TMPRSS15, SCG3, PPP1R12A and LRP1) were amongst the top differentially expressed proteins between MPR and non-MPR patients at baseline.

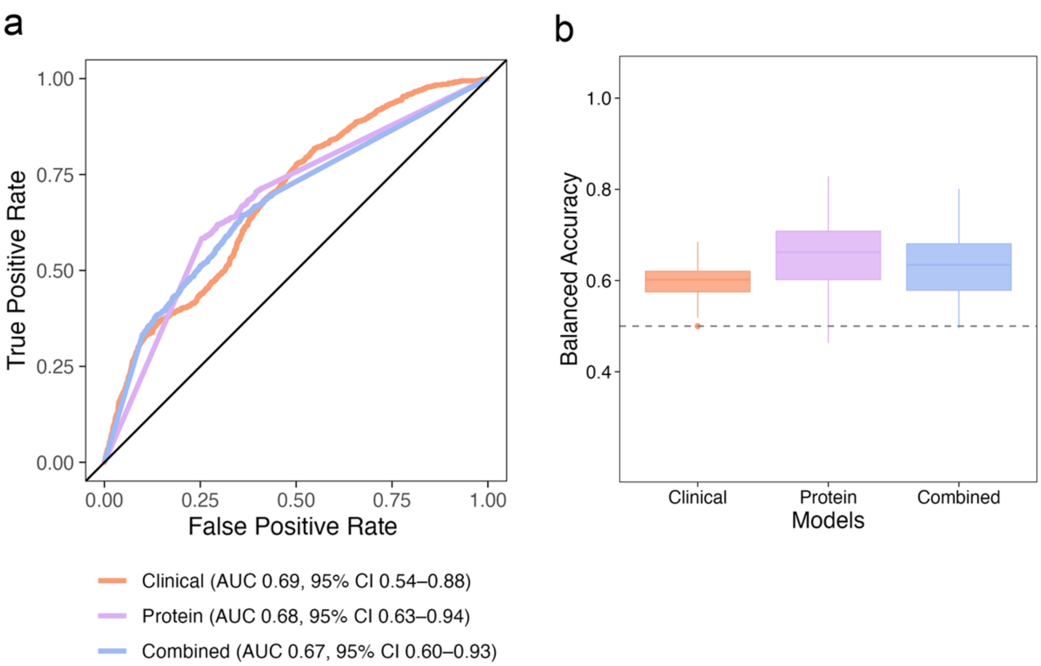

Figure S6. Predictive performance of GLM using clinical variables, protein signature or the combination.

(a) Receiver operating characteristic curves and (b) balanced accuracy plot showing the predictive performance of the GLM using clinical variables (age, sex and stage), the 10-protein IFNγ signature identified from feature selection on the 188 IFNγ-associated proteins at baseline, or the combined model (clinical and protein). ROC curve AUC values with 95% confidence estimated from averaged predictions per sample shown.

Supplementary Tables

Table S1. Differentially expressed proteins (*P*-value < 0.05) between patients with major pathological response (MPR) vs non-MPR at baseline, EDT and pre-surgery.

**Baseline**							
**Protein**	**MPR**	**Non-MPR**	**Log_2 fold change**	**Average expression**	**t**	***P*-value**	**Adjusted *P*-value**
**CCL7**	0.34	-0.79	1.13	0.005	5.17	$6.63x10^{-6}$	$4.65x10^{-3}$
**HAVCR1**	0.60	-0.40	1.01	0.29	3.98	$2.76x10^{-4}$	$6.32x10^{-2}$
**MMP12**	0.15	-0.54	0.69	-0.07	3.92	$3.28x10^{-4}$	$6.32x10^{-2}$
**ICA1**	0.04	0.65	-0.61	0.23	-3.89	$3.60x10^{-4}$	$6.32x10^{-2}$
**TMPRSS15**	0.44	-0.69	1.13	0.09	3.48	$1.20x10^{-3}$	$1.69x10^{-1}$
**SCG3**	-0.19	0.28	-0.47	-0.05	-3.24	$2.39x10^{-3}$	$2.57x10^{-1}$
**TREM2**	0.16	-0.45	0.60	-0.03	3.18	$2.85x10^{-3}$	$2.57x10^{-1}$
**PRSS8**	0.28	-0.35	0.62	0.08	3.10	$3.53x10^{-3}$	$2.57x10^{-1}$
**ADM**	0.06	-0.22	0.28	-0.02	3.09	$3.60x10^{-3}$	$2.57x10^{-1}$
**PPP1R12A**	0.09	0.45	-0.36	0.20	-3.08	$3.67x10^{-3}$	$2.57x10^{-1}$
**BCR**	0.01	0.47	-0.46	0.15	-2.97	$4.93x10^{-3}$	$3.13x10^{-1}$
**TNFAIP8**	-0.56	0.53	-1.09	-0.22	-2.93	$5.54x10^{-3}$	$3.13x10^{-1}$
**LRP1**	0.12	-0.13	0.25	0.04	2.90	$5.94x10^{-3}$	$3.13x10^{-1}$
**IL17F**	0.18	-0.60	0.78	-0.06	2.87	$6.42x10^{-3}$	$3.13x10^{-1}$
**AXIN1**	0.07	0.60	-0.53	0.23	-2.86	$6.68x10^{-3}$	$3.13x10^{-1}$
**IKBKG**	0.01	0.48	-0.47	0.15	-2.78	$8.19x10^{-3}$	$3.54x10^{-1}$
**CXCL8**	0.15	-0.27	0.41	0.02	2.75	$8.81x10^{-3}$	$3.54x10^{-1}$
**SH2B3**	-0.04	0.95	-0.98	0.26	-2.72	$9.51x10^{-3}$	$3.54x10^{-1}$
**MLN**	0.28	-0.66	0.94	-0.01	2.72	$9.57x10^{-3}$	$3.54x10^{-1}$
**PPP1R9B**	-0.12	0.31	-0.44	0.01	-2.65	$1.15x10^{-2}$	$3.62x10^{-1}$
**CD40**	0.14	-0.14	0.29	0.05	2.64	$1.18x10^{-2}$	$3.62x10^{-1}$
**AMBP**	0.07	-0.08	0.15	0.02	2.63	$1.20x10^{-2}$	$3.62x10^{-1}$
**CCL8**	0.02	-0.41	0.43	-0.11	2.63	$1.21x10^{-2}$	$3.62x10^{-1}$

**IL7**	0.13	-0.38	0.52	-0.03	2.62	$1.24x10^{-2}$	$3.62x10^{-1}$
**RRM2**	0.12	-0.37	0.49	-0.03	2.58	$1.35x10^{-2}$	$3.79x10^{-1}$
**PLXNA4**	0.33	-0.04	0.37	0.22	2.55	$1.45x10^{-2}$	$3.92x10^{-1}$
**BANK1**	0.01	0.48	-0.47	0.15	-2.47	$1.78x10^{-2}$	$4.47x10^{-1}$
**FABP9**	-0.28	0.28	-0.56	-0.11	-2.43	$1.94x10^{-2}$	$4.47x10^{-1}$
**LYPD8**	0.04	-0.67	0.71	-0.18	2.43	$1.95x10^{-2}$	$4.47x10^{-1}$
**STX16**	-0.12	0.50	-0.63	0.07	-2.42	$2.01x10^{-2}$	$4.47x10^{-1}$
**HCLS1**	-0.15	0.29	-0.43	-0.01	-2.37	$2.25x10^{-2}$	$4.47x10^{-1}$
**CXCL17**	0.26	-0.19	0.46	0.12	2.36	$2.31x10^{-2}$	$4.47x10^{-1}$
**OMG**	-0.32	0.40	-0.73	-0.10	-2.36	$2.34x10^{-2}$	$4.47x10^{-1}$
**IL15RA**	-0.13	-0.47	0.34	-0.24	2.34	$2.44x10^{-2}$	$4.47x10^{-1}$
**REG4**	0.23	-0.14	0.37	0.12	2.31	$2.63x10^{-2}$	$4.47x10^{-1}$
**DAG1**	0.06	-0.20	0.26	-0.02	2.30	$2.63x10^{-2}$	$4.47x10^{-1}$
**SORD**	0.39	0.02	0.38	0.28	2.30	$2.64x10^{-2}$	$4.47x10^{-1}$
**IL18**	0.13	-0.23	0.36	0.02	2.30	$2.66x10^{-2}$	$4.47x10^{-1}$
**FAM3B**	0.16	-0.07	0.24	0.09	2.29	$2.75x10^{-2}$	$4.47x10^{-1}$
**NCK2**	0.12	0.45	-0.33	0.22	-2.28	$2.78x10^{-2}$	$4.47x10^{-1}$
**EDA2R**	0.18	-0.19	0.37	0.07	2.28	$2.79x10^{-2}$	$4.47x10^{-1}$
**MYO9B**	0.04	0.28	-0.24	0.12	-2.28	$2.80x10^{-2}$	$4.47x10^{-1}$
**CEP20**	0.17	-0.20	0.37	0.06	2.28	$2.80x10^{-2}$	$4.47x10^{-1}$
**EPO**	0.23	-0.38	0.60	0.04	2.27	$2.87x10^{-2}$	$4.47x10^{-1}$
**MSRA**	0.08	0.25	-0.17	0.13	-2.26	$2.90x10^{-2}$	$4.47x10^{-1}$
**PTPN6**	-0.05	0.54	-0.59	0.13	-2.26	$2.95x10^{-2}$	$4.47x10^{-1}$
**VPS53**	0.03	0.31	-0.29	0.12	-2.24	$3.09x10^{-2}$	$4.47x10^{-1}$
**RAD23B**	0.15	-0.13	0.28	0.07	2.23	$3.10x10^{-2}$	$4.47x10^{-1}$
**CLIP2**	0.07	0.51	-0.44	0.20	-2.21	$3.25x10^{-2}$	$4.47x10^{-1}$
**CPE**	-0.38	0.11	-0.49	-0.23	-2.20	$3.33x10^{-2}$	$4.47x10^{-1}$
**BGN**	0.04	-0.44	0.47	-0.11	2.19	$3.45x10^{-2}$	$4.47x10^{-1}$
**LAT2**	-0.04	0.21	-0.25	0.04	-2.18	$3.53x10^{-2}$	$4.47x10^{-1}$
**LYN**	0.10	-0.26	0.36	-0.01	2.16	$3.66x10^{-2}$	$4.47x10^{-1}$
**SORCS2**	0.14	-0.20	0.34	0.04	2.14	$3.82x10^{-2}$	$4.47x10^{-1}$

**NELL2**	-0.19	0.10	-0.30	-0.10	-2.14	3.83x10^{-2}	4.47x10^{-1}
**CD84**	0.03	-0.16	0.18	-0.03	2.14	3.84x10^{-2}	4.47x10^{-1}
**MME**	0.46	-0.10	0.56	0.29	2.14	3.85x10^{-2}	4.47x10^{-1}
**SEZ6L**	-0.11	0.12	-0.23	-0.04	-2.13	3.96x10^{-2}	4.47x10^{-1}
**SAMD9L**	-0.02	0.23	-0.26	0.06	-2.12	3.98x10^{-2}	4.47x10^{-1}
**STX4**	0.45	0.09	0.36	0.34	2.12	4.00x10^{-2}	4.47x10^{-1}
**F2R**	0.11	-0.21	0.32	0.01	2.12	4.03x10^{-2}	4.47x10^{-1}
**CLEC4C**	0.10	0.61	-0.51	0.26	-2.12	4.04x10^{-2}	4.47x10^{-1}
**DCBLD2**	0.23	-0.14	0.37	0.11	2.11	4.06x10^{-2}	4.47x10^{-1}
**SLC39A5**	0.19	-0.18	0.38	0.08	2.11	4.07x10^{-2}	4.47x10^{-1}
**FLT3LG**	0.04	-0.37	0.41	-0.08	2.11	4.14x10^{-2}	4.47x10^{-1}
**HTRA2**	0.11	-0.07	0.19	0.06	2.09	4.29x10^{-2}	4.51x10^{-1}
**MPIG6B**	0.33	-0.04	0.37	0.22	2.09	4.33x10^{-2}	4.51x10^{-1}
**CCN1**	0.24	-0.10	0.34	0.13	2.08	4.37x10^{-2}	4.51x10^{-1}
**CCL3**	-0.06	-0.41	0.35	-0.17	2.06	4.54x10^{-2}	4.58x10^{-1}
**GZMB**	0.15	-0.43	0.58	-0.03	2.06	4.57x10^{-2}	4.58x10^{-1}
**FGF21**	0.58	-0.50	1.08	0.25	2.04	4.81x10^{-2}	4.69x10^{-1}
**CXCL9**	-0.51	-1.19	0.69	-0.72	2.04	4.81x10^{-2}	4.69x10^{-1}
**TBL1X**	-0.07	0.23	-0.29	0.02	-2.03	4.92x10^{-2}	4.69x10^{-1}
**CA14**	-0.09	0.19	-0.28	-0.01	-2.02	4.98x10^{-2}	4.69x10^{-1}

**EDT**							
**Protein**	**MPR**	**Non-MPR**	**Log$_2$ fold change**	**Average expression**	**t**	***P*-value**	**Adjusted *P*-value**
**HAVCR1**	0.40	-0.86	1.27	-0.04	5.05	2.53x10^{-5}	1.78x10^{-2}
**TREM2**	0.31	-0.61	0.92	-0.01	3.82	6.99x10^{-4}	1.92x10^{-1}
**BCR**	-0.18	0.35	-0.53	0.00	-3.75	8.22x10^{-4}	1.92x10^{-1}
**ENPP7**	0.52	-0.71	1.23	0.10	3.33	2.51x10^{-3}	4.15x10^{-1}
**MMP12**	0.52	-0.39	0.91	0.20	3.26	2.95x10^{-3}	4.15x10^{-1}
**DECR1**	-0.09	0.50	-0.59	0.12	-2.95	6.47x10^{-3}	5.96x10^{-1}

**CLEC4C**	0.02	0.86	-0.83	0.31	-2.92	6.97×10^{-3}	5.96×10^{-1}
**VPS53**	-0.06	0.26	-0.32	0.05	-2.83	8.61×10^{-3}	5.96×10^{-1}
**ADGRG1**	0.94	-0.16	1.09	0.56	2.82	8.86×10^{-3}	5.96×10^{-1}
**FLT3LG**	0.24	-0.37	0.61	0.03	2.77	9.81×10^{-3}	5.96×10^{-1}
**MEPE**	-0.12	0.15	-0.27	-0.02	-2.77	9.91×10^{-3}	5.96×10^{-1}
**LAT2**	-0.11	0.26	-0.37	0.02	-2.63	1.38×10^{-2}	5.96×10^{-1}
**MLN**	-0.04	-1.20	1.16	-0.44	2.63	1.38×10^{-2}	5.96×10^{-1}
**MME**	0.51	-0.28	0.79	0.24	2.63	1.39×10^{-2}	5.96×10^{-1}
**CXCL9**	1.01	0.17	0.84	0.72	2.59	1.53×10^{-2}	5.96×10^{-1}
**ANKRD54**	0.40	0.91	-0.51	0.58	-2.54	1.69×10^{-2}	5.96×10^{-1}
**BANK1**	0.06	0.52	-0.46	0.22	-2.47	1.99×10^{-2}	5.96×10^{-1}
**SPRY2**	-0.22	0.34	-0.55	-0.02	-2.46	2.04×10^{-2}	5.96×10^{-1}
**KLRD1**	0.32	-0.24	0.56	0.12	2.45	2.08×10^{-2}	5.96×10^{-1}
**MYO9B**	-0.14	0.20	-0.34	-0.02	-2.44	2.13×10^{-2}	5.96×10^{-1}
**ICA1**	-0.09	0.55	-0.65	0.13	-2.44	2.13×10^{-2}	5.96×10^{-1}
**LRIG1**	-0.13	0.32	-0.45	0.03	-2.44	2.14×10^{-2}	5.96×10^{-1}
**SLAMF8**	0.83	0.18	0.65	0.61	2.41	2.27×10^{-2}	5.96×10^{-1}
**TPT1**	-0.59	1.75	-2.34	0.22	-2.39	2.37×10^{-2}	5.96×10^{-1}
**MAPK9**	0.06	-0.18	0.24	-0.02	2.39	2.38×10^{-2}	5.96×10^{-1}
**DDX58**	0.35	0.71	-0.36	0.47	-2.39	2.38×10^{-2}	5.96×10^{-1}
**SCG3**	-0.15	0.27	-0.43	-0.01	-2.38	2.44×10^{-2}	5.96×10^{-1}
**DGKZ**	-0.15	0.73	-0.88	0.15	-2.38	2.46×10^{-2}	5.96×10^{-1}
**PRSS8**	0.12	-0.37	0.49	-0.05	2.35	2.63×10^{-2}	5.96×10^{-1}
**MMP10**	0.23	-0.23	0.46	0.07	2.35	2.64×10^{-2}	5.96×10^{-1}
**CLIP2**	-0.19	0.23	-0.43	-0.05	-2.35	2.64×10^{-2}	5.96×10^{-1}
**FABP9**	-0.18	0.38	-0.56	0.01	-2.33	2.72×10^{-2}	5.96×10^{-1}
**KLRB1**	0.31	-0.07	0.38	0.18	2.32	2.80×10^{-2}	5.96×10^{-1}
**SPINK6**	0.32	-0.01	0.33	0.20	2.29	2.96×10^{-2}	6.12×10^{-1}
**FSTL3**	-0.03	-0.31	0.28	-0.12	2.27	3.12×10^{-2}	6.26×10^{-1}
**RP2**	-0.11	0.20	-0.31	0.00	-2.25	3.24×10^{-2}	6.31×10^{-1}
**BACH1**	-0.13	0.19	-0.32	-0.02	-2.24	3.33×10^{-2}	6.33×10^{-1}

**DSG4**	-0.22	0.31	-0.53	-0.04	-2.23	$3.44x10^{-2}$	$6.36x10^{-1}$
**C4BPB**	-0.06	0.28	-0.34	0.05	-2.18	$3.84x10^{-2}$	$6.80x10^{-1}$
**AIF1**	-0.29	0.52	-0.80	-0.01	-2.14	$4.13x10^{-2}$	$6.80x10^{-1}$
**FIS1**	0.14	-0.16	0.30	0.04	2.14	$4.18x10^{-2}$	$6.80x10^{-1}$
**CCL7**	0.63	-0.02	0.65	0.40	2.13	$4.20x10^{-2}$	$6.80x10^{-1}$
**PVALB**	0.33	-0.31	0.64	0.11	2.13	$4.23x10^{-2}$	$6.80x10^{-1}$
**LGALS7_ LGALS7B**	0.05	-0.31	0.36	-0.07	2.10	$4.51x10^{-2}$	$6.80x10^{-1}$
**IFNLR1**	-0.07	0.33	-0.40	0.07	-2.09	$4.56x10^{-2}$	$6.80x10^{-1}$
**ACTN4**	-0.41	0.19	-0.60	-0.21	-2.09	$4.63x10^{-2}$	$6.80x10^{-1}$
**IL17C**	0.20	0.85	-0.65	0.42	-2.08	$4.66x10^{-2}$	$6.80x10^{-1}$
**CNPY4**	-0.04	-0.31	0.28	-0.13	2.08	$4.72x10^{-2}$	$6.80x10^{-1}$
**ATOX1**	0.28	-0.06	0.34	0.16	2.07	$4.74x10^{-2}$	$6.80x10^{-1}$

**Pre-surgery**							
**Protein**	**MPR**	**Non-MPR**	**Log_2 fold change**	**Average expression**	**t**	***P*-value**	**Adjusted *P*- value**
**MIA**	-0.30	0.69	-0.98	0.005	-4.04	$2.33x10^{-4}$	$1.55x10^{-1}$
**HAVCR1**	0.53	-0.56	1.09	0.20	3.82	$4.42x10^{-4}$	$1.55x10^{-1}$
**RABGAP1L**	0.33	0.83	-0.51	0.48	-3.44	$1.34x10^{-3}$	$3.15x10^{-1}$
**AIF1**	-0.20	0.64	-0.84	0.06	-3.33	$1.85x10^{-3}$	$3.25x10^{-1}$
**PRSS8**	0.37	-0.23	0.60	0.19	3.23	$2.43x10^{-3}$	$3.42x10^{-1}$
**ICA1**	0.10	0.68	-0.58	0.28	-3.08	$3.65x10^{-3}$	$4.27x10^{-1}$
**MLN**	-0.02	-1.16	1.14	-0.37	2.75	$8.78x10^{-3}$	$8.53x10^{-1}$
**COL9A1**	0.00	0.73	-0.73	0.23	-2.71	$9.72x10^{-3}$	$8.53x10^{-1}$
**AMBN**	-0.19	0.07	-0.27	-0.11	-2.57	$1.38x10^{-2}$	$9.25x10^{-1}$
**KDR**	-0.13	0.13	-0.26	-0.05	-2.57	$1.38x10^{-2}$	$9.25x10^{-1}$
**SCG3**	-0.10	0.31	-0.40	0.03	-2.52	$1.56x10^{-2}$	$9.25x10^{-1}$
**LHB**	0.09	1.43	-1.34	0.50	-2.52	$1.58x10^{-2}$	$9.25x10^{-1}$

**MEGF10**	-0.22	0.10	-0.33	-0.12	-2.43	1.94×10^{-2}	9.72×10^{-1}
**CXCL17**	0.15	-0.38	0.53	-0.01	2.23	3.13×10^{-2}	9.72×10^{-1}
**HCLS1**	-0.01	0.32	-0.33	0.09	-2.22	3.18×10^{-2}	9.72×10^{-1}
**SEZ6L2**	-0.23	0.08	-0.32	-0.13	-2.21	3.30×10^{-2}	9.72×10^{-1}
**MME**	0.26	-0.36	0.63	0.07	2.12	4.02×10^{-2}	9.72×10^{-1}
**INPP1**	-0.12	0.22	-0.33	-0.01	-2.08	4.38×10^{-2}	9.72×10^{-1}
**LYPD3**	-0.11	0.18	-0.29	-0.02	-2.06	4.59×10^{-2}	9.72×10^{-1}
**ATP5IF1**	0.36	-0.08	0.44	0.22	2.05	4.70×10^{-2}	9.72×10^{-1}
**PSPN**	0.37	-0.74	1.11	0.03	2.05	4.71×10^{-2}	9.72×10^{-1}

Differential expression results are based on the normalized NPX values using RUVg. Log_2 fold change values represent the difference in protein expression between MPR and non-MPR patients.

Table S2. 50 upstream regulators from the Ingenuity Pathway Analysis (IPA) with activation z-score > 2 and *P*-value < 0.05.

Upstream regulator	Activation z-score	*P*-value	Target molecules in dataset
**APP**	3.19	8.62×10^{-6}	AXIN1, CCL3, CCL7, CD40, CXCL8, CXCL9, IKBKG, IL18, IL7, LRP1, MME, MMP12, TREM2
**poly rI:rC-RNA**	3.12	2.09×10^{-4}	CCL3, CCL7, CCL8, CD40, CXCL8, CXCL9, GZMB, IL15RA, IL18, LYN
**IL1B**	2.92	2.38×10^{-9}	ADM, BGN, CCL3, CCL7, CCL8, CD40, CLIP2, CXCL8, CXCL9, EPO, GZMB, IL15RA, IL17F, IL18, IL7, LYN, MMP12, SAMD9L, TREM2
**TLR4**	2.91	2.65×10^{-9}	ADM, CCL3, CCL8, CCN1, CD40, CXCL8, CXCL9, FLT3LG, IL15RA, IL17F, IL18, TREM2
**AKT (family)**	2.80	2.40×10^{-5}	ADM, CCN1, CXCL8, EPO, IL17F, MME, MMP12, RRM2
**PDGF-BB (complex)**	2.79	6.15×10^{-6}	ADM, CCL7, CCL8, CCN1, CXCL8, DCBLD2, EPO, MMP12
IFNG	*2.79*	5.47×10^{-9}	*ADM, CCL3, CCL7, CCL8, CD40, CLIP2, CXCL17, CXCL8, CXCL9, F2R, GZMB, IL15RA, IL18, IL7, LAT2, LYN, MMP12, PRSS8, PTPN6, SAMD9L, TREM2*
**MYD88**	2.78	5.43×10^{-6}	CCN1, CD40, CXCL8, CXCL9, GZMB, IL17F, IL18, IL7
**IL6**	2.78	8.07×10^{-7}	ADM, AXIN1, BGN, CCL7, CD40, CXCL8, CXCL9, EPO, GZMB, IL17F, IL7, MMP12, RRM2
**CEBPB**	2.74	3.89×10^{-4}	ADM, CCL3, CCL7, CXCL8, IL7, LYN, PRSS8, RRM2

**P38 MAPK (family)**	2.73	$1.28x10^{-5}$	BGN, CCL7, CCL8, CD40, CXCL8, CXCL9, FGF21, MMP12
**Lipo-polysaccharide**	2.70	$2.22x10^{-11}$	ADM, BANK1, BGN, CCL3, CCL7, CCL8, CCN1, CD40, CXCL8, CXCL9, DAG1, DCBLD2, EDA2R, F2R, FGF21, GZMB, HCLS1, IL15RA, IL17F, IL18, IL7, LRP1, LYN, MME, MMP12, PTPN6, RRM2, SAMD9L, STX4, TNFAIP8, TREM2
**RIGI**	2.63	$8.58x10^{-9}$	CCL3, CCL7, CCL8, CXCL8, CXCL9, IL15RA, IL18, MME
**HIF1A**	2.60	$6.31x10^{-5}$	ADM, BGN, CCN1, CXCL8, CXCL9, EPO, IL17F, IL7, MMP12
**Cigarette smoke**	2.59	$1.40x10^{-5}$	ADM, CCN1, CXCL8, IL18, LRP1, MMP12, TREM2
**STING1**	2.58	$2.30x10^{-6}$	CCL7, CXCL8, CXCL9, IL15RA, IL18, MME, MMP12
**Imiquimod**	2.57	$3.04x10^{-8}$	CCL3, CD40, CXCL17, CXCL8, CXCL9, DAG1, IL17F, IL18, RAD23B
**IL18**	2.45	$2.45x10^{-5}$	CCL3, CD40, CXCL8, CXCL9, GZMB, IL18
**IL2**	2.43	$2.15x10^{-7}$	CCL3, CD40, CXCL8, CXCL9, FLT3LG, GZMB, IL17F, IL18, MME, PTPN6, RRM2, SORD, TNFAIP8
**IL12 (complex)**	2.41	$2.72x10^{-5}$	CCL3, CXCL8, CXCL9, GZMB, IL18, RAD23B
**IL27**	2.41	$1.21x10^{-9}$	CCL3, CCL7, CCL8, CXCL8, CXCL9, GZMB, IL15RA, IL17F, IL18, IL7
**KLF6**	2.40	$1.17x10^{-4}$	ADM, CXCL8, DCBLD2, IL15RA, LYN, MMP12

**Interferon alpha (family)**	2.40	1.08×10^{-9}	BCR, CCL3, CCL7, CD40, CXCL8, CXCL9, DAG1, F2R, GZMB, IL15RA, IL7, PTPN6, SAMD9L, TREM2
**VEGF (family)**	2.38	1.63×10^{-5}	CCL7, CCN1, CD40, CXCL8, DCBLD2, EPO, IL15RA, IL18, LYN
**ERK1/2 (family)**	2.37	3.41×10^{-4}	CCL3, CCL7, CCL8, CD40, CXCL8, MMP12
**F2**	2.37	1.39×10^{-4}	CCL8, CCN1, CXCL8, EPO, F2R, IL15RA
**TLR9**	2.37	6.79×10^{-5}	CCL3, CD40, CXCL8, CXCL9, FLT3LG, IL18
**CD3 (complex)**	2.36	1.93×10^{-3}	CCL3, CXCL8, GZMB, IL15RA, LRP1, LYN, RAD23B
**STAT1**	2.30	3.48×10^{-5}	CCL3, CD40, CXCL8, CXCL9, GZMB, IL15RA, IL18, SAMD9L
**MAVS**	2.24	2.48×10^{-6}	CCL7, CCL8, CXCL8, IL15RA, IL18, MME
**NFKB (complex)**	2.22	3.18×10^{-8}	ADM, CCL3, CCL7, CCL8, CCN1, CD40, CLIP2, CXCL8, CXCL9, IL15RA, IL18, STX4, TNFAIP8
**RNase1**	2.22	2.71×10^{-9}	CCL3, CCL7, CCL8, CXCL9, IL7
**5-O-mycolyl-beta-araf-(1->2)-5-O-mycolyl-alpha-araf-(1->1')-glycerol**	2.22	3.80×10^{-6}	CCL7, CCL8, CD40, CXCL8, CXCL9
**Concanavalin A**	2.22	4.76×10^{-5}	CCL3, CXCL8, CXCL9, IL17F, IL18
**TYROBP**	2.21	6.69×10^{-12}	CCL3, CCL7, CD40, CD84, CXCL8, IL18, SH2B3, TREM2

**E. coli B5 lipopolysaccharide**	2.21	8.43×10^{-4}	CD40, CXCL8, IL15RA, IL18, IL7
**RNase2**	2.20	3.60×10^{-8}	CCL3, CCL7, CCL8, CXCL9, IL7
**TNF (family)**	2.20	1.01×10^{-4}	CCL3, CCL8, CD40, CXCL8, CXCL9, SAMD9L
**CCR2**	2.20	1.53×10^{-5}	BGN, CD40, CXCL9, IL17F, IL18
**MIF**	2.19	4.14×10^{-6}	CD84, CXCL8, F2R, GZMB, IL15RA, IL7
**Histamine**	2.19	2.05×10^{-5}	CCL3, CD40, CXCL8, GZMB, IL18
**Peptidoglycan**	2.19	1.60×10^{-5}	CCL3, CCL8, CD40, CXCL8, IL18
**TBK1**	2.19	1.89×10^{-5}	CD40, CXCL8, CXCL9, IL15RA, IL18
**Insulin (family)**	2.18	1.17×10^{-3}	CA14, CXCL8, EPO, FGF21, IL18, LRP1, RRM2, STX4
**TNF**	2.17	1.63×10^{-9}	ADM, AXIN1, BCR, BGN, CCL3, CCL7, CCL8, CCN1, CD40, CXCL8, CXCL9, DAG1, DCBLD2, EPO, IL15RA, IL18, IL7, LRP1, LYN, MMP12, MYO9B, RRM2, SAMD9L, TNFAIP8, TREM2
**CD40**	2.17	3.22×10^{-6}	CCL3, CD40, CXCL8, CXCL9, FLT3LG, GZMB, IL15RA, IL18
**Palmitic acid**	2.17	1.99×10^{-2}	CPE, CXCL8, FGF21, IL18, RAD23B
**AP1 (complex)**	2.15	1.61×10^{-5}	CCL3, CD40, CXCL8, GZMB, MMP12, RRM2
**TLR3**	2.15	1.24×10^{-4}	CCL3, CD40, CXCL8, CXCL9, FLT3LG, GZMB
**Adavosertib**	2.09	2.00×10^{-8}	CCL7, CCL8, CXCL8, IL15RA, IL18, MME, RRM2

Upstream regulators were inferred using Ingenuity Pathway Analysis (IPA), which predicts activation or inhibition of transcription factors, cytokines, or other regulatory molecules based on observed expression changes in downstream targets. Activation z-score reflects the predicted activation state of each upstream regulator based on the

direction of expression changes in downstream targets. *P*-value indicates the statistical significance of overlap between observed and predicted downstream targets, calculated using Fisher's exact test. The 21 downstream targets of IFNγ (IFNG) are italicised.

Table S3. 188 IFNγ-associated proteins derived from the Ingenuity Pathway Analysis (IPA) IFNG downstream targets and MSigDB HALLMARK_INTERFERON_GAMMA_RESPONSE pathway.

ADAMTS8	CDKN1A	GFAP	IL7	PNPT1
ADGRG1	CEACAM1	GMPR	IRAK1	POLR2F
ADM	CEACAM5	GZMA	ITGA6	PRKCQ
AGER	CEP85	GZMB	ITGAV	PRSS8
AGRN	CLIP2	HBEGF	ITGB7	PTPN6
AIF1	CRIM1	HLA-DRA	JUN	PTX3
ANGPTL4	CRLF1	HSPA1A	KDR	RARRES1
AREG	CSF1	ICOSLG	KRT19	SAMD9L
ARG1	CSF3	IFNG	KYNU	SCLY
BACH1	CTSC	IFNGR1	LAG3	SIGLEC10
BANK1	CTSV	IGF1R	LAMP3	SLAMF1
BTN3A2	CXADR	IKZF2	LAP3	SLAMF7
C1QA	CXCL1	IL10	LAT2	SMAD1
CALB1	CXCL10	IL10RA	LGALS9	SPON1
CASP2	CXCL12	IL11	LIFR	TGFB1
CASP8	CXCL17	IL12B	LY75	TGFBR2
CCL11	CXCL3	IL12RB1	LYN	TIMP3
CCL13	CXCL6	IL13	MANF	TLR3
CCL17	CXCL8	IL13RA1	MERTK	TNF
CCL20	CXCL9	IL15	METAP2	TNFRSF11A
CCL22	DCTN2	IL15RA	MMP1	TNFRSF11B
CCL23	DDX58	IL17A	MMP10	TNFRSF12A
CCL25	DECR1	IL17D	MMP12	TNFRSF14
CCL26	DLL1	IL17RB	NAMPT	TNFSF10
CCL28	EGF	IL18	NBN	TNFSF11
CCL3	ENTPD2	IL18R1	NCF2	TNFSF12
CCL4	ERBB2	IL1B	NDUFS6	TNFSF13
CCL7	ESM1	IL1RL2	NFKBIE	TP53

CCL8	F2R	IL1RN	NTF4	TRAF2
CCN2	F3	IL2	OGFR	TREM2
CD200	FASLG	IL22RA1	OSM	TRIM21
CD27	FCGR2B	IL2RB	PAPPA	VEGFA
CD276	FGF2	IL32	PDCD1	VEGFC
CD33	FLT1	IL3RA	PDCD1LG2	WNT9A
CD38	FLT4	IL4	PDGFB	XCL1
CD4	FOXO1	IL4R	PDGFC	ZBTB16
CD40	FURIN	IL5	PLA2G4A	
CD83	FXN	IL6	PLAUR	

Table S4. The 10 proteins in the baseline IFNγ signature differentially expressed (*P*-value < 0.05) between patients with major pathological response (MPR) vs non-MPR.

Protein	UniProt ID	MPR	Non-MPR	Log_2 fold change	*P*-value	Adjusted *P*-value
**CCL7**	P80098	0.34	-0.79	1.13	6.63×10^{-6}	4.65×10^{-3}
**MMP12**	P39900	0.15	-0.54	0.69	3.28×10^{-4}	6.32×10^{-2}
**ADM**	P35318	0.06	-0.22	0.28	3.60×10^{-3}	2.57×10^{-1}
**TREM2**	Q9NZC2	0.16	-0.45	0.60	2.85×10^{-3}	2.57×10^{-1}
**PRSS8**	Q16651	0.28	-0.35	0.62	3.53×10^{-3}	2.57×10^{-1}
**CD40**	P25942	0.14	-0.14	0.29	1.18×10^{-2}	3.62×10^{-1}
**CXCL8**	P10145	0.15	-0.27	0.41	8.81×10^{-3}	3.54×10^{-1}
**CCL8**	P80075	0.02	-0.41	0.43	1.21×10^{-2}	3.62×10^{-1}
**IL7**	P13232	0.13	-0.38	0.52	1.24×10^{-2}	3.62×10^{-1}
**IL15RA**	Q13261	-0.13	-0.47	0.34	2.44×10^{-2}	4.47×10^{-1}

Table S5. The classification performance metrics of the baseline 10-protein IFNγ signature

Metric	AUC, 95% confidence interval
Sensitivity	0.74 (95% CI 0.55–0.87)
Specificity	0.75 (95% CI 0.47–0.91)
Positive predictive value (PPV)	0.87 (95% CI 0.68–0.95)
Negative predictive value (NPV)	0.56 (95% CI 0.33–0.77)
